# Ideal Dose of Iron in Multiple Micronutrient Supplement: A Narrative Review of Evidence

**DOI:** 10.7759/cureus.28688

**Published:** 2022-09-02

**Authors:** Anjusha Ranjith, Surabhi Puri, Kashish Vohra, Areeba Khanam, Mohan Bairwa, Ravneet Kaur, Kapil Yadav

**Affiliations:** 1 Epidemiology and Public Health, All India Institute of Medical Sciences, New Delhi, IND

**Keywords:** iron deficiency, hemoglobin, anemia, ifa, iron folic-acid, mms, unimmap, multiple micronutrient supplement, pregnancy

## Abstract

Anemia is a significant public health problem in low- and middle-income countries (LMICs). The co-existence of other micronutrient deficiencies and iron deficiency among pregnant women may be the reason for the inability to control anemia through iron and folic acid (IFA) supplementation. Multiple micronutrient supplementation (MMS) in pregnancy may help to overcome this problem. However, the recent World Health Organization (WHO) guidelines on MMS supplementation in pregnancy raised concerns regarding the adequacy of a 30mg iron dose in the MMS supplements in LMICs. The review summarized the literature to answer this question. Though most studies showed a comparable effect of MMS with 30mg iron and IFA with 60mg iron on maternal anemia outcomes, anemia persisted in the third trimester in both groups. There is a need to consider the use of a higher iron dose in MMS, especially in LMICs, to combat the problem of anemia, alongside correcting other micronutrient deficiencies.

## Introduction and background

Anemia in pregnancy is a significant public health problem worldwide. The World Health Organization (WHO) 2019 estimated that 32 million pregnant women globally were anemic, constituting 36.5% of all pregnant women [1]. Various global and national guidelines have tried to combat this problem by supplementing iron and folic acid (IFA) in pregnancy. The WHO guidelines in 2016 recommended the use of IFA supplements containing 30-60mg of elemental iron and 400 µg folic acid daily throughout pregnancy [2]. This recommendation favored using 60mg of iron in the IFA supplements in areas where the prevalence of anemia was more than forty percent.

Multiple micronutrient deficiencies are prevalent among pregnant women, especially in low- and middle-income countries (LMICs) [3-5]. Women in these areas primarily consume nutritionally inadequate diets and enter pregnancy with poor micronutrient statuses, and the increased demands further compound this during pregnancy. Multiple micronutrient supplementation in pregnancy is associated with reduced babies born at small-for-gestational age, low-birth weight, and pre-term [6-8]. Given the high prevalence of micronutrient deficiencies among pregnant women and the accumulating evidence on the beneficial effects of multiple micronutrient supplements (MMS) during pregnancy on birth outcomes, an update of the WHO antenatal care guidelines in 2020 recommended the use of "antenatal multiple micronutrient supplements that include iron and folic acid, in the context of rigorous research" [9]. This recommendation was based on studies using MMS containing 13-15 micronutrients (including United Nations International Multiple Micronutrient Antenatal Preparation - UNIMMAP), conducted mainly in LMICs.

The conditional recommendation in the WHO 2020 guidelines was because of the advocacy of a 60mg daily iron dose in pregnant populations with a severe public health problem (anemia prevalence of 40% or higher). Most MMS preparations contained 30mg of iron, and the implications of switching to 30mg of iron in high anemia prevalence areas were unclear. Hence, this review aims to summarize the available literature on the adequacy of 30mg iron in MMS compared to 60mg iron in IFA to control anemia and iron deficiency among pregnant women in LMICs. The review also explores the need for a higher dose of iron in multiple micronutrient supplements.

## Review

Methods

PubMed and Google Scholar were searched for relevant studies. Randomized controlled trials (RCTs) in pregnant women with no known comorbidities, comparing MMS containing 30mg iron with IFA containing 60mg iron, and systematic reviews comparing MMS with iron with/without folic acid in pregnancy were included for narrative synthesis. Only those studies reporting anemia, hemoglobin, or iron deficiency were included. For RCTs, inclusion in the study was restricted to those conducted in LMICs. Only studies published in the English language before 15 June 2022 were included. No restrictions were placed on the period during which the studies were conducted. The following keywords were used to identify studies - pregnancy, multiple micronutrient supplements, iron folic acid, anemia, hemoglobin, and iron deficiency.

Result

A double-blinded randomized controlled trial in Nepal by Osrin (2005) [10] randomized pregnant women to receive either UNIMMAP or IFA (60mg iron and 400 µg folic acid). The authors found that the prevalence of anemia decreased by 12% and 10%, and hemoglobin levels increased by 0.3 ± 1.41 g/dl and 0.3 ± 1.50 g/dl in the MMS and IFA groups, respectively. The findings were replicated in Burkina Faso in a factorial double-blinded randomized controlled trial in pregnant women conducted by Roberfroid (2008) [11]. In this study, one of the components of the factorial design was the comparison of UNIMMAP with IFA (60mg iron and 400 µg folic acid). Roberfroid found that the maternal hemoglobin levels in the third trimester, after directly observed supplement intake, were comparable (mean difference= 0.03 g/dl).

The findings from other studies also showed the absence of significant differences in anemia and hemoglobin levels between the two supplement groups. A double-blinded cluster randomized controlled trial having UNIMMAP vs. IFA (60mg iron and 400 µg folic acid) comparison in pregnant women was conducted by Zeng (2009) [12] in rural China. This study showed comparable effects of the supplements on hemoglobin levels (mean difference= 0.17 ± 1.45 g/dl) and maternal anemia prevalence (43.1% and 45.1% in MMS and IFA groups, respectively) in the third trimester. Another study, a single-blinded cluster randomized controlled trial by Sunawang (2009) [13], was conducted in Indonesia, in which clusters were randomized to receive either UNIMMAP or IFA (60mg iron and 0.25mg folic acid). The study found that the changes in the prevalence of anemia were comparable in the two arms of the trial.

Similarly, a factorial RCT in Bangladesh by Persson (2012) [14], in which the micronutrient supplementation was double-blinded, compared UNIMMAP with IFA (60mg iron & 400 μg folic acid) in two arms of the trial. Persson found comparable changes in hemoglobin levels in the two arms (-0.1 ± 1.25 g/dl in the IFA group and -0.24 ± 1.25 g/dl in the UNIMMAP group).

A cluster randomized controlled trial in Pakistan by Bhutta (2009) [15] allocated pregnant women to receive either UNIMMAP or IFA (60mg iron and 400 µg folic acid). Bhutta reported reductions in anemia prevalence of 5.8% and 8.3% and changes in hemoglobin levels from baseline to end line of 0.2 ± 1.59 g/dl and 0.1 ± 1.55 g/dl in the MMS and IFA groups, respectively. However, the rise in serum ferritin level was more significant with IFA, which contained a higher dose of iron (change in ferritin of 15.3 ± 32.46 ng/ml with MMS and 20.9 ± 32.59 ng/ml with IFA) (Table 1).

**Table 1:** Studies comparing MMS containing 30mg iron and IFA containing 60 mg iron for effects on maternal anemia and hemoglobin levels The table summarizes the randomized controlled trials in pregnant women comparing multiple micronutrient supplements containing 30mg iron and iron folic acid containing 60mg iron. UNIMMAP contained 30mg of elemental iron, 400 μg of folic acid, 800 μg of vitamin A, 1.4mg of vitamin B1, 1.4mg of vitamin B2, 18mg niacin, 1.9mg of vitamin B6, 2.6 μg of vitamin B12, 70mg vitamin C, 5 μg vitamin D, 10mg vitamin E, 2mg copper, 150 μg iodine, 65 μg selenium, and 15mg zinc. UNIMMAP- United Nations International Multiple Micronutrient Antenatal Preparation; MMS- Multiple Micronutrient Supplement; IFA- Iron and Folic acid; MD- Mean Difference

S. No.	Study	Site	Intervention arm	Control arm	Findings
1.	Osrin (2005 [10]	Nepal	UNIMMAP	IFA (60 mg iron and 400 µg folic acid)	Anemia prevalence decreased by 12% and 10%, and hemoglobin levels increased by 0.3 ± 1.41 g/dl and 0.3 ± 1.50 g/dl in MMS and IFA groups, respectively.
2.	Roberfroid (2008) [11]	Burkina Faso	UNIMMAP	IFA (60 mg iron and 400 µg folic acid)	Comparable maternal hemoglobin in the third trimester (MD: 0.03g/dl).
3.	Zeng (2009) [12]	China	UNIMMAP	IFA (60 mg iron and 400 µg folic acid)	Comparable effects on hemoglobin levels (MD: 0.17+1.45 g/dl) and maternal anemia (43.1% and 45.1%) in the third trimester.
4.	Sunawang (2009) [13]	Indonesia	UNIMMAP	IFA (60 mg iron and 0.25 mg folic acid)	Comparable changes in anemia prevalence.
5.	Persson (2012) [14]	Bangladesh	UNIMMAP	IFA (60 mg iron and 400 µg folic acid)	Comparable changes in hemoglobin levels (-0.1 ± 1.25 g/dl in the IFA group, and -0.24 ± 1.25 g/dl in the MMS group).
6.	Bhutta (2009) [15]	Pakistan	UNIMMAP	IFA (60 mg iron and 400 µg folic acid)	Comparable reductions in anemia prevalence (5.8% and 8.3%), and changes in hemoglobin levels (0.2+1.59 g/dl and 0.1+1.55 g/dl) in the MMS and IFA groups, respectively. A rise in serum ferritin is greater with IFA (15.3 ± 32.46 ng/ml with MMS and 20.9 ± 32.59 ng/ml with IFA).

A systematic review and meta-analysis by Haider (2011) [16] pooled data from four RCTs that compared MMS with IFA supplementation in pregnancy. The study found comparable effects of the supplements on maternal anemia in the third trimester (RR: 1.03, 95% CI: 0.87 to 1.22). Similar results were found by Bhutta (2012) [17] by pooling data from seven RCTs. Bhutta found a relative risk of 1.03 (95% CI: 0.94 to 1.12) for the outcome of maternal anemia in the third trimester, with moderate outcome-specific quality. The authors, using data from five RCTs, reported a mean difference in hemoglobin levels in the third trimester of -0.01 (95% CI: -0.08 to 0.06) g/dl, with low outcome-specific quality.

Keats (2019) [6], pooling data from nine RCTs comparing MMS and iron with/ without folic acid in pregnant women, found comparable effects of the supplements on maternal anemia in the third trimester (RR: 1.04, 95% CI: 0.94 to 1.15). Oh C (2020) [8], in another meta-analysis, pooled data from studies comparing MMS and iron with/ without folic acid in pregnancy. Sixteen RCTs provided data for the outcome of maternal anemia in the third trimester and reported a pooled relative risk of 1.02 (95% CI: 0.95 to 1.10), with high-quality evidence. The authors also reported a relative risk of 1.12 (95% CI: 0.62 to 2.02), with very low-quality evidence, for iron deficiency anemia in the third trimester, pooling four RCTs.

A systematic review by Gomes F (2022) [18] compared third-trimester maternal anemia and iron status with MMS and IFA supplementation in pregnancy. The authors included eleven RCTs for this comparison and found that MMS and IFA had similar effects on third-trimester hemoglobin levels, anemia, and iron deficiency anemia. Sub-group analyses by the daily iron dose in the supplements and the total supplemental iron intake showed no differences between MMS and IFA groups for the three outcomes. For the comparison of MMS with 30mg iron and IFA with 60mg iron, the mean difference in hemoglobin levels in the third trimester was -0.26 g/dl (95% CI: -1.41 to 0.89, 4 trials, 3882 participants). Similarly, the study found a relative risk of 0.99 (95% CI: 0.92 to 1.07; 5 trials, 4677 participants) for maternal anemia and 1.31 (95% CI: 0.66 to 2.60, 2 trials, 590 participants) for iron deficiency anemia in the third trimester (Table 2).

**Table 2:** Systematic reviews and meta-analyses comparing MMS and iron (with/without folic acid) for effects on maternal anemia and hemoglobin levels The table contains a summary of the systematic reviews and meta-analyses comparing MMS and iron with/without folic-acid in pregnancy. RCTs- Randomized Controlled Trials; MMS- Multiple Micronutrient Supplement; IFA- Iron and Folic acid; RR- Relative Risk; MD- Mean Difference; CI- Confidence Interval

S. No.	Study	Included studies	Intervention arm	Control arm	Findings
1.	Haider (2011) [16]	4 RCTs	MMS	IFA	Comparable effects on maternal anemia in the third trimester (RR: 1.03, 95% CI: 0.87 to 1.22)
2.	Bhutta (2012) [17]	7 RCTs	MMS	IFA	Comparable effects on maternal anemia (RR: 1.03, 95% CI: 0.94 to 1.12), and hemoglobin levels (MD: -0.01, 95% CI: -0.08 to 0.06) in third trimester.
3.	Keats (2019) [6]	9 RCTs	MMS	Iron with/without folic acid	Comparable maternal anemia in the third trimester (RR: 1.04, 95% CI: 0.94 to 1.15)
4.	Oh C (2020) [8]	16 RCTs	MMS	Iron with/without folic acid	Comparable effects on maternal anemia (RR: 1.02, 95% CI: 0.95 to 1.10) and iron deficiency anemia (RR: 1.12, 95% CI: 0.62 to 2.02) in the third trimester.
5.	Gomes F (2022) [18]		MMS with 30mg iron	IFA with 60mg iron	Comparable risk of anemia (RR: 0.99, 95% CI: 0.92 to 1.07, 5 RCTs), hemoglobin levels in third trimester (MD: -0.26 g/dl, 95% CI: -1.41 to 0.89, 4 RCTs), and risk of iron deficiency anemia (RR:1.31, 95% CI: 0.66 to 2.60, 2 RCTs).

Discussion

Overall, multiple micronutrient supplements in pregnancy showed an effect of comparable magnitude on maternal anemia and hemoglobin levels compared to the traditional iron folic-acid supplements. This evidence was supported by numerous systematic reviews, including that by Haider (2011) [16], Bhutta (2012) [17], Keats (2019 [6], and Oh C (2020) [8]. However, the findings of these systematic reviews cannot be taken at face value due to the high heterogeneity in most of them, as a consequence of the inclusion of studies using different doses and combinations of iron in the two supplements. A large fraction of the included studies used a lower dose of iron in the MMS than in the IFA supplement. This included studies using the UNIMMAP preparation containing 30mg of iron. Most studies used 60mg of iron-containing IFA in the control arm, as most countries, especially LMICs, use 60mg of iron in their routine IFA supplements. These systematic reviews also failed to show the relative benefits of the different doses of iron used in the MMS supplements.

Randomized controlled trials are comparing MMS containing 30mg iron with IFA containing 60mg iron demonstrated the absence of significant differences in the effects of the supplements on anemia and hemoglobin levels. Osrin (2005) [10] in Nepal, Sunawang (2009) [13] in Indonesia, and Persson (2012) [14] in Bangladesh found comparable changes in anemia prevalence and hemoglobin levels with supplementation. Similarly, Roberfroid (2008) [11] in Burkina Faso and Zeng (2009) [12] in China showed a comparable prevalence of anemia and comparable hemoglobin levels at the end of supplementation. The baseline prevalence of anemia among pregnant women was high in all the studies mentioned above, and it was 38% and 43.3% at baseline in studies by Osrin (2005) [10] and Roberfroid (2008) [11], respectively. A pilot study ahead of the Zeng (2009) study [12] had shown 57% anemia prevalence among pregnant women in the third trimester in the study area. Previous studies had estimated anemia prevalence of 40% and 34.7% among pregnant women in Indonesia and Bangladesh, respectively, where Sunawang (2009) [19] and Persson (2012) [20] studies were conducted. Despite the high prevalence of anemia, the lower dose of iron in the MMS supplements produced comparable effects on anemia and hemoglobin levels as traditional IFA supplements.

The presence of other micronutrients like vitamin A, C, and B-complex vitamins in MMS, in addition to iron, may have enhanced the absorption and assimilation of iron from the MMS supplements. Moreover, the presence of these hematinic vitamins in MMS may have helped correct their deficiencies and, thereby, the associated anemia. The evidence for this may be seen in trials by Schulze (2019) [21], Osrin (2005) [10], Sunawang (2009) [13], and a meta-analysis by Allen (2009) [22], that showed an increase in the serum levels of various micronutrients like vitamin B12, vitamin A, vitamin D, and vitamin E; and a reduction in the prevalence of their deficiencies with MMS supplementation. This may have resulted in the two supplements' analogous effects on anemia and hemoglobin levels.

Most of the studies comparing a lower dose of iron in MMS with IFA assessed the effects of these supplements on anemia and hemoglobin levels, which were found to be comparable. However, anemia persisted in the third trimester in both groups despite supplementation, highlighting that neither of the supplements adequately controlled anemia in pregnancy [12,13,15]. This may hint at the need for exploring higher doses of iron in these supplements, especially in high-burden populations.

The cluster randomized controlled trial in Pakistan by Bhutta (2009) [15] reported reductions in anemia prevalence and changes in hemoglobin levels from baseline to end line of comparable magnitude in the MMS and IFA groups. The baseline prevalence of anemia (49%) and iron deficiency (35%) in this study population were high. Though MMS with a lower dose of iron had comparable effects on anemia and hemoglobin levels as IFA, this lower dose was insufficient to replenish the iron stores. This was evidenced by the more significant increase in serum ferritin levels with IFA, which contained a higher dose of iron. This may further emphasize the inability of low-dose iron in MMS to correct iron deficiency in populations with a high baseline prevalence of iron deficiency.

With the WHO raising concerns regarding the use of a lower dose of iron in MMS compared to that in IFA, the systematic review by Gomes F (2022) [18] attempted to explore the effects of the supplements on maternal anemia outcomes in LMICs. This study compared the effects of MMS and IFA supplementation in pregnancy on third-trimester maternal anemia and iron status indicators. The doses of iron in both the supplements examined these outcomes in subgroups. None of the subgroups showed differences between MMS and IFA for outcomes of maternal anemia, maternal hemoglobin levels, or maternal iron deficiency anemia. Specifically, MMS containing 30mg iron, when compared with IFA containing 60mg iron, was not found to be associated with an increased risk of anemia or lower hemoglobin levels. The five trials that provided data for comparing maternal anemia had a high prevalence of anemia at baseline. There was also no evidence of an increased risk of iron deficiency anemia despite the lower dose of iron in MMS. However, only two studies provided data for this outcome, so the confidence in this estimate is low.

The ideal dose of iron in the MMS supplements is currently debated. An iron dose below 20mg was found to significantly increase iron deficiency and iron deficiency anemia and result in lower hemoglobin and ferritin levels in an iron dose-response study for anemia prophylaxis in pregnancy (Milman 2005) [23]. This was supported by evidence from two randomized controlled trials - by Adu-Afarwuah S (2017) [24] in Ghana and Jorgensen (2018) [25] in Malawi comparing MMS with 20mg of iron and 17 micronutrients in one intervention arm against a control arm supplemented with IFA (60mg iron and 400 µg folic acid). In both these studies, the MMS group had lower hemoglobin levels, a higher prevalence of anemia, and higher iron deficiency at the end of supplementation.

The aforementioned iron dose-response study for anemia prophylaxis in pregnancy reported that a 40mg daily iron dose was adequate to prevent iron deficiency in 90% of the women and iron deficiency anemia in 95% of the women. This was demonstrated by comparable effects on iron status indicators (hemoglobin and ferritin levels) with 40mg, 60mg, and 80mg iron doses [23]. However, a clear dose-response relationship between increasing mean hemoglobin levels with increasing iron dose (hemoglobin at the end of treatment was 111 ± 13 g/l with 20mg daily iron, 114 ± 11 g/l with 40mg daily iron, and 119 ± 12 g/l with 80mg daily iron, p=0.006) was demonstrated by Zhou (2007) [26] in Australia, for treating anemia in pregnancy. Since over 40% of the pregnant women in LMICs are anemic, the prophylactic dose of iron, as recommended by Milman et al. [23], may be inadequate to correct the anemia in these women. Also, Zhou et al. [26] showed that at the end of treatment, the regression coefficient for hemoglobin concentration was 0.12 ± 0.04 g/l (p= 0.001) and that for ferritin concentration was 1.0 ± 1.0 µg/l (p<0.001). Evidence for this was also seen in the Bhutta (2009) study [15], where 30mg of iron in MMS, compared to 60mg in IFA, was inadequate to replenish the iron stores.

Though MMS containing a lower dose of iron may be at par with IFA containing 60mg iron for controlling anemia, neither supplement showed a satisfactory response in anemia prevalence. Also, this lower iron dose may not be adequate for the control of iron deficiency in pregnancy. If 60mg of iron is used in MMS, the additional micronutrients present in MMS may further enhance iron absorption, over and above that from IFA with the same iron dose. They may help outperform the traditional IFA supplements in their effects on anemia and hemoglobin level. Though evidence for this better performance is not evident from the existing literature (Gomes 2022; RR for anemia: 1.06, 95% CI: 0.82 to 1.37; MD in hemoglobin: -0.68 g/dl, 95% CI: -3.56 to 2.20; RR for iron deficiency anemia: 1.18, 95% CI: 0.94, 1.48, for the comparison of MMS and IFA with 60mg iron), this was based on only three studies [18]. Also, the micronutrient composition of the MMS used varied between these three studies, and one of the studies did not use MMS with 13-15 micronutrients as recommended by the WHO.

Hence, there is a need to explore using a higher dose of iron in the MMS supplements to tackle the problem of anemia and iron deficiency, especially in LMICs. This higher dose of iron may also address the subgroup differences that indicate lower neonatal mortality with IFA containing 60mg iron, as compared with MMS, seen in the WHO 2020 study [9].

Currently, the WHO guidelines recommend using 120mg of iron for treating anemia in pregnant women diagnosed with anemia until their hemoglobin concentration rises to normal [2]. This raises another question about the prophylactic and therapeutic doses of iron that needs to be used alongside MMS supplementation. It is evident from the above discussion that the prophylactic iron dose in MMS may require a rise. However, how the therapeutic dose would vary when used along with MMS is a subject to be explored.

## Conclusions

This review summarized the evidence on using 30mg of iron in multiple micronutrient supplements in pregnancy. The available literature showed that MMS containing 30mg iron was comparable to IFA containing 60mg iron for its effects on maternal anemia outcomes. Lack of power and high heterogeneity among the included studies may have been the reason for the comparable results. The prevalence of anemia was high at the end of supplementation, and the consequence of the supplementation on iron deficiency was equivocal. Considering the high prevalence of anemia and iron deficiency in LMICs, 30mg of iron may be insufficient in pregnancy. A higher dose of iron in the MMS supplement than that present in the UNIMMAP preparation should be considered. Well-designed randomized controlled trials comparing 30mg and 60mg iron in MMS may be prudent to explore the ideal dose of iron in these supplements that can mitigate the high burden of anemia.

## Appendices

PubMed search strategy:

#1 "Dietary Supplements"[Mesh] OR “multiple micronutrient*”[TiAb] OR “multiple micronutrient supplement*”[TiAb] OR micronutrient*[TiAb] OR MMS[TiAb] OR MMN[TiAb] OR UNIMMAP[TiAb] OR “dietary supplement*”[TiAb] OR multivitamin[TiAb]

#2 "Anemia"[Mesh] OR Anemia[TiAb] OR anaemia[TiAb] OR hemoglobin[TiAb] OR haemoglobin[TiAb] OR hematocrit[TiAb] OR “iron status”[TiAb] OR “iron defecienc*”[TiAb]

#3 "Pregnancy"[Mesh] OR Pregnanc*[TiAb] OR pregnant*[TiAb] OR “pregnant women”[TiAb] OR prenatal[TiAb] OR antenatal[TiAb]

#4 “clinical trial”[Publication Type] OR “clinical trials as topic”[MeSH Terms] OR “clinical trial”[All Fields]

#1 AND #2 AND #3 AND #4

Google scholar search strategy:

With all of the words: micronutrient, pregnancy

With the exact phrase: multiple micronutrient

With at least one of the words: anemia, anaemia, hemoglobin, haemoglobin, hematocrit, iron status, iron deficiency
